# Muscular Force Patterns during Level Walking in ACL-Deficient Patients with a Concomitant Medial Meniscus Tear

**DOI:** 10.1155/2019/7921785

**Published:** 2019-05-05

**Authors:** Hongshi Huang, Wei Yin, Shuang Ren, Yuanyuan Yu, Si Zhang, Qiguo Rong, Yingfang Ao

**Affiliations:** ^1^Beijing Key Laboratory of Sports Injuries, Institute of Sports Medicine, Peking University Third Hospital, 100191 Beijing, China; ^2^Department of Mechanics and Engineering Science, College of Engineering, Peking University, 100871 Beijing, China

## Abstract

**Background:**

The abnormal knee joint motion patterns caused by anterior cruciate ligament (ACL) deficiency are thought to be associated with articular cartilage degeneration. High rates of meniscus tear combined with ACL rupture are observed, and these knees suffer a higher risk of early cartilage degeneration.

**Research Question:**

This study investigated lower limb muscular force patterns of ACL-deficient knees with a concomitant medial meniscus tear.

**Methods:**

12 volunteers and 22 patients were recruited, including 12 patients with isolated ACL deficiency (ACLD) and 10 ACL-deficient patients with a concomitant medial meniscus tear (ACLDM). Level walking data at a self-selected speed were collected before surgery. Then, a musculoskeletal dynamic analysis system, AnyBody, was applied to simulate tibiofemoral flexion moments and muscle forces.

**Results:**

Our results indicate that the tibiofemoral peak flexion and extension moments in ACLDM patients are significantly lower than in controls. The rectus femoris force in ACLDM patients was significantly lower than in isolated ACL-deficient patients and the controls during mid and terminal stance phase, while no significant difference was found in hamstring and vastus force. Additionally, the gastrocnemius force in ACL-deficient patients both with and without a medial meniscus tear was lower than in controls during mid-stance phase.

**Significance:**

The ACLDM patients had lower peak tibiofemoral flexion moment, lower gastrocnemius force in mid-stance phase, and lower rectus femoris force during the mid and terminal stance phase. These results may help clinicians to better understand the muscle function and gait pattern in ACL-deficient patients with a concomitant medial meniscus tear.

## 1. Introduction

Anterior cruciate ligament (ACL) rupture accounts for 20% percent of sport-related knee injuries [1]. Since the ACL plays an important role in maintaining knee stability, ACL-injured patients have abnormal knee motion during level walking, which has been reported by many researchers [2–7]. However, the muscular force patterns in ACL-injured patients are unclear. Berchuck et al. found an abnormal gait pattern during the stance phase of gait using gait analysis in ACL-deficient individuals, which was called “quadriceps avoidance gait” [7]. Quadriceps serves as a knee extensor [8]; the decreased quadriceps activity may result in a smaller knee extension moment. But later studies pointed out that this gait pattern was not commonly found in ACL-deficient patients [2, 3]. The “hamstring facilitation” strategy [9] may also cause the lower extension moment because the hamstring is a flexor which opposes the extensors. An isolated increase in hamstring activity would also generate a smaller knee extension moment.

Additionally, it is well known that the meniscus plays an important role in load-bearing distribution and maintaining knee stability [10]. Recent research, which was performed by the Swedish National Knee Ligament Registry, showed that 40% of ACL ruptures were combined with meniscus injures [11]. An ACL-deficient knee with a concomitant meniscus tear has a higher risk of early cartilage degeneration [6, 12–15]. Ahn et al. found that a longitudinal tear of the posterior horn of the medial meniscus in an ACL-deficient knee can alter the knee kinematics, especially anterior–posterior tibial translation [12]. In a gait analysis of ACL-deficient patients with and without meniscus injury, Harato et al. showed that ACL-deficient patients with severe meniscus injury exhibited more abnormal gait mechanics than did patients with an isolated ACL deficiency, particularly in axial knee excursion during the stance phase, which was significantly larger in the affected knee with a concomitant meniscus injury compared to the unaffected knee [6]. Additional studies showed that a concomitant meniscus tear altered knee kinematics in different ways depending on the pattern of the meniscus tear [13, 14]. The key to restoring normal movement is to restore muscle strength affected by injury, which casts the importance on better understanding of the muscular force patterns that patients adapt to. Understanding the muscle patterns of ACL-deficient knees with a concomitant meniscus tear before reconstructive surgery is necessary to help clinicians develop more efficient pre-habilitation programs, which may allow for earlier ACL reconstruction.

While concomitant lateral meniscus injury occurs concurrently with ACL injury, concomitant medial meniscus tear usually follows ACL injury [16]; therefore, focusing on concomitant medial meniscus tear may provide insights to better understanding on meniscus injury after ACL deficiency. The overall goal of our study was to investigate the effect of concomitant medial meniscus tear on knee flexion moment and muscle patterns during level walking. We hypothesized that, during the stance phase, ACL-deficient patients with a concomitant medial meniscus tear would exhibit more abnormal muscle patterns than would patients without a medial meniscus tear, especially the quadriceps and hamstrings.

## 2. Methods

### 2.1. Subjects

We selected some of the participants as subjects analyzed in our research. The selection criteria were to ensure that factors in each group were not statistically different from one another (*P* > 0.05), including age, body mass index (BMI), gender, and walking pace. Based on this principle, 34 subjects were selected from our database. Written informed consent was attained from all participants. The ethical approval was obtained from the university's ethics committee. A total of 12 patients with isolated ACL deficiency were allocated to the ACLD group, and 10 ACL-deficient patients with a concomitant meniscus tear were allocated to the ACLDM group. No patient suffered severe cartilage injury. The cartilage defects in patients were less than grade II according to the Outerbridge system [17], which was confirmed by practiced clinicians after surgery. In terms of medial meniscus tears, many kinds of tears were found in the medial meniscus, including posterior horn meniscus root tears and horizontal and longitudinal tears. However, we did not specify the details of meniscus tear type because of the limited number of participants. Twelve volunteers who had no history of musculoskeletal injury or surgery in the lower extremities, and who had no measurable ligamentous instability on clinical examination, made up the control group. All details of those subjects are shown in Table 1. All participants are male.

### 2.2. Instrumentation and Data Collection

Gait data were collected before ACL reconstruction surgery. The measurements were performed by an eight-camera VICON system (Vicon MX, Oxford Metrics, UK) and two force plates (AMTI, Watertown, Massachusetts, USA; 1000 Hz). Retroreflective markers were placed according to the biomechanical model of Helen Hayes [18]. Participants were asked to walk on a level floor (approximately 10 meters long) at a self-selected speed. All patients had no pain feeling during walking.

Preoperative side-to-side difference in anterior laxity was measured by a KT 2000 arthrometer (MRS, KneelaxIII, Holland) at 132 N force under general anesthesia, and preoperative isokinetic muscle moment of knee extensor and flexor in the affected and unaffected knees were measured by an isokinetic dynamometer (Con-Trex MJ, Germany) at the angular velocity of 60°/s. The average peak flexion and extension moments were normalized by body mass (Nm/kg).

### 2.3. Modeling

Motion capture data and force plate data were imported into a commercial musculoskeletal modeling program (AnyBody, Aalborg, Denmark) [19]. Generically scaled models of each participants were created based on the anthropometric data of Horsman et al. [20]. The Twente Lower Extremity Model (TLEM), which consists of 159 muscles and 6 joint degrees of freedom, was employed in our research. The body length and mass were used to scale soft tissues.

Before the inverse dynamic analysis process, the kinematics were simulated by applying an optimization method that defines the position of each segment in relation to the measurement markers. After accomplishing kinematics optimization, inverse dynamic simulation was performed with a min/max recruitment solver [21]. The tibiofemoral joint (TFJ) was modeled as a hinge joint due to the soft tissue artifact error [22].

The AnyMuscleModel, which has been proven to be reasonable in gait simulation, was employed as the muscle model in our study. Our results included the muscle forces and flexion moment. All of the TFJ flexion moment and muscle forces were averaged for every subject from three trials of gait data. All simulation results were resampled to 101 values corresponding to 100% of the stance phase of gait (0% to 60% of the gait cycle) by cubic spline interpolation.

### 2.4. Statistical Analysis

The averaged muscle forces were normalized by body weight (N/kg), while the TFJ flexion moments were normalized by body weight and height (Nm/(kg^∗^m)).

Considering the limited number of subjects, power analysis for ANOVA designs was applied to evaluating the significance of our results. Simulated TFJ flexion moments, and muscle forces at every moment of stance phase between the three groups were analyzed by applying a one-way analysis of variance method. The peak value of each parameter was analyzed by the same method (for parameters that had biphasic waveforms, peak 1 and 2, sorted by occurring moment, were analyzed separately). A post hoc pairwise comparison between each two groups was then performed. All statistical analyses were performed using Matlab (Matlab 2014a, Natick, Massachusetts USA). *P* values of <0.05 were considered significant.

## 3. Results

### 3.1. Knee Anterior Laxity and Muscle Strength

The results of knee anterior laxity and muscle strength are shown in Table 2. No significant difference was found between ACLD and ACLDM groups.

### 3.2. Tibiofemoral Flexion Moments and Muscular Force Patterns

**Figure 1:**
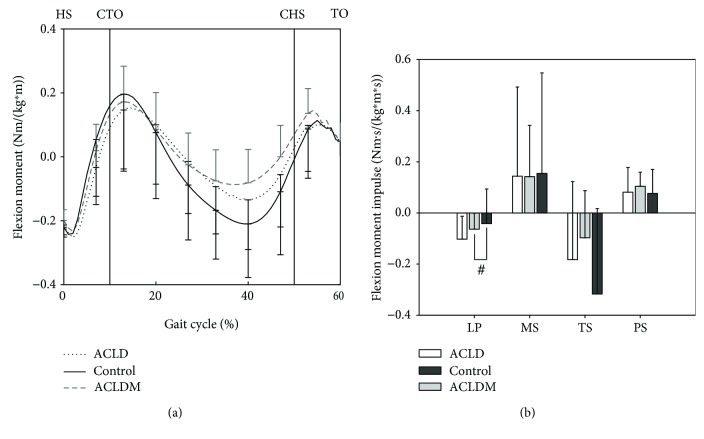
Simulated tibiofemoral flexion moment (a) and flexion moment impulse (b) during stance phase. LP: loading response phase (0-10% of gait); MS: mid-stance phase (10-30% of gait); TS: terminal stance phase (30-50% of gait); PS: pre-swing phase (50-60% of gait); HS: heel strike; CTO: contralateral toe-off; CHS: contralateral heel strike; TO: toe-off. Significant difference: ^#^*P* < 0.05 between ACLDM and control groups.

**Figure 2:**
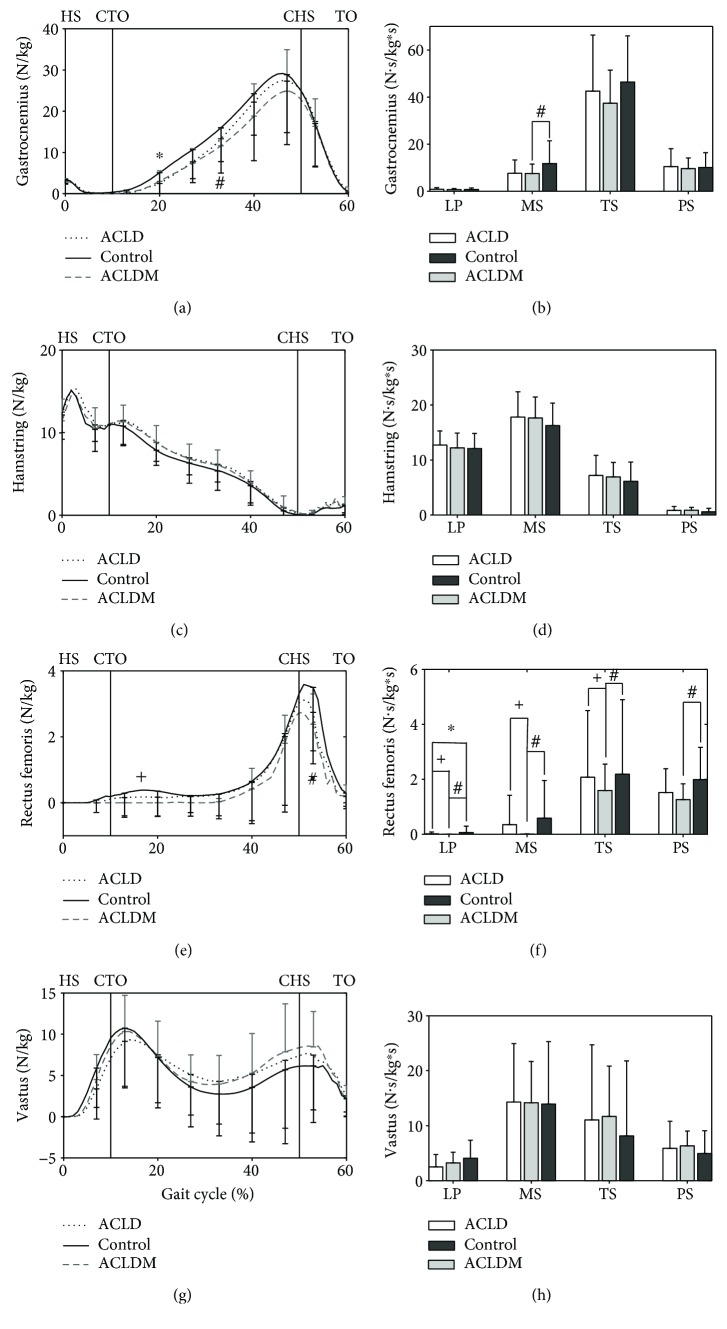
Simulated muscular force patterns (left) and force impulses (right) during stance phase. LP: loading response phase (0-10% of gait); MS: mid-stance phase (10-30% of gait); TS: terminal stance phase (30-50% of gait); PS: pre-swing phase (50-60% of gait); HS: heel strike; CTO: contralateral toe-off; CHS: contralateral heel strike; TO: toe-off. Significant difference: ^∗^*P* < 0.05 between ACLD and control groups. ^#^*P* < 0.05 between ACLDM and control groups. ^+^*P* < 0.05 between ACLD and ACLDM groups.

The simulated tibiofemoral flexion moments and muscle forces during stance phase are shown in Figures 1 and 2. The muscles included the gastrocnemius, rectus femoris, hamstring, and vastus (vastus medialis, vastus lateralis, and vastus intermedius). Moreover, the impulses of each parameter during the four phases (loading response phase, mid-stance phase, terminal stance phase, and pre-swing phase) [23] were also analyzed.

#### 3.2.1. Tibiofemoral Flexion Moment

Similar trends were observed between each group in flexion moment waveforms and moment impulses, as shown in Figure 1.

No significant differences between ACLD and control or between ACLD and ACLDM were found. However, significant differences between the ACLDM and control groups were observed during 44-54% of gait. In this period, the extension moment in the ACLDM group was lower than in the control group. Moreover, the peak flexion and extension moments in the ACLDM groups were significantly lower than in the control group, as shown in Table 3. Interestingly, though there were no significant differences between each group in the loading response phase, the flexion moment impulse during this phase in the ACLDM group was significantly different than in the control group.

#### 3.2.2. Muscular Force Patterns

All muscle forces and muscle force impulses had a similar shape between each group, as shown in Figure 2.

For the gastrocnemius (GAS) (Figures 2(a) and 2(b)), the peak muscle force occurred at the end of the terminal stance phase where no significant differences were found. However, during the mid-stance phase, the GAS force in the ACLD group was significantly different than in the control group (8-22% of gait), and that in the ACLDM group was significantly different than in the control group (9-35% of gait). Moreover, the GAS force impulse in the ACLDM group during the mid-stance phase was significantly smaller than in the control group. Power analysis showed that our results achieve 98% power to detect the differences among three groups.

In terms of hamstring (HAM) (Figures 2(c) and 2(d)) and vastus (VAS) (Figures 2(g) and 2(h)), no significant differences were found between the groups in muscle forces or muscle force impulses. However, for the rectus femoris (RF) (Figures 2(e) and 2(f)), the power of our results reached 86% and a significant difference between the ACLD and ACLDM groups was found during 7-41% of gait. The RF force in the ACLDM group was also significantly different than in the control group during 7-53% of gait. In those periods, the RF force in the ACLDM group was smaller than in the ACLD and control groups. Moreover, the peak RF force in the ACLDM group was significantly lower than in the ACLD and control groups (Table 3). Apart from the significant differences found in muscular force patterns, many significant differences were observed in RF muscle impulses. In the loading response phase, the RF force impulse was significantly different in each group, and the impulse in the ACLDM group was significantly different than in the other two groups in the mid-stance and terminal stance phases. The RF force impulse in the ACLDM group was also significantly different from the control group in the pre-swing phase. Additionally, during all four phases, the RF force impulses in the ACLDM group were the lowest.

## 4. Discussion

The results supported our hypothesis that the gait in ACL-deficient patients with a medial meniscus tear has more abnormal kinetics and muscular force patterns as compared with those without a meniscus tear: (1) during the middle and terminal stance phases, the rectus femoris force in ACLDM patients was significantly lower than in the ACLD group and the control group; (2) during the mid-stance phase, the gastrocnemius force in ACLDM and ACLD patients was lower than in the control group; and (3) the peak flexion and extension moments in ACLDM patients were lower than in the control group.

Many previous findings support that joint unloading, not overloading, may be associated with the cascade of early degenerative changes of the knee [24–26]. In our study, lower peak flexion and extension moments were found in ACLDM patients. And the lower flexion moment impulse during the loading response phase confirmed the joint unloading in ACLDM patients. However, those parameters were not significantly different between ACLD patients and the controls. This may explain the higher incidence of cartilage degeneration in ACLDM patients.

The “quadriceps avoidance gait” has been identified and discussed as an adaptive mechanism in ACL-deficient patients in many studies [3, 4, 7, 27–29]. This gait pattern was first reported by Berchuck et al. [7]. After ACL injury, decreased quadriceps activity is thought to reduce the peak knee flexion moment during normal gait in order to compensate for the decreased resistance to anterior tibial translation [7]. However, other studies found that this pattern was not common in ACL-deficient patients [3, 28, 30]. Notably, a recent study found that post-injury knee stability can influence the quadriceps activity in ACLD patients [8, 31]. In our results, all patients presented positive Lachman test, which means that they had unstable knee stability. Moreover, the patients with isolated ACL deficiency had a lower RF impulse than the controls during the loading response phase and ACL-deficient patients with a concomitant meniscus tear had lower RF force than the controls during almost all of the stance phase. In the meantime, the peak flexion moment in the ACLDM group was significantly lower than in the control group, which indicated lower quadriceps activity. However, no significant differences were found between each group in the HAM force. Thus, quadriceps inhibition might be the main factor contributing to a reduced flexion moment.

In terms of extension moment, which reflects the net activity of the knee flexors, reduced extension moment indicates either lower HAM activity or greater quadriceps activity, or a combination of the above [31]. We found a lower extension moment in ACLDM patients in our study. In contrast, there were no significant differences between the HAM and VAS between the ACLDM and control groups. Meanwhile, the RF, another part of the quadriceps, had lower activity during the terminal stance phase. It appears that our predicted muscle activity did not exactly match the results for extension moment. However, considering the limited number of ACLDM subjects in our research and the higher averaged VAS force in the ACLDM group during the terminal stance phase, a larger sample size may lead to more reasonable results. Thus, further work is needed to determine whether the VAS force during terminal stance is significantly larger than in the controls.

The gastrocnemius muscle is also a knee flexor. Though its role in knee biomechanics and on the ACL remains incompletely understood, some researchers believe that it serves as an ACL antagonist, pushing the tibia anteriorly and increasing the strain of the ACL [32, 33]. In our results, the GAS forces in ACL-deficient patients both with and without a concomitant medial meniscus tear were significantly lower than in the controls during the mid-stance phase, and the GAS impulse in ACLDM patients during mid-stance was also significantly smaller than in the controls. Thus, the reduced GAS activity might be interpreted as an adaptation in ACL-deficient patients to reduce anterior tibial translation, which was similar to the quadriceps avoidance gait.

There were some limitations in our study. First, we had a limited number of subjects. We had only 10 ACL-deficient patients with a concomitant medial meniscus tear and 12 isolated ACL-deficient patients. Meanwhile, though we controlled the walking pace of subjects, the standard deviations in age, BMI, and time since injury in the three groups were relatively large, which may have resulted in the large standard deviations in every predicted knee joint parameter and lower reliability of the statistical results. The variance in our results was not simply due to interindividual variability, but was generated by the error involved with external marker motion analysis and the musculoskeletal model. The main modeling limitation was that the TFJ was simulated as a hinge joint, while in reality the TFJ can rotate and translate in all three planes. However, considering that the soft tissue artifact error in the thigh and leg was the highest and the musculoskeletal model was susceptible to error, the simplification of segments and ignoring soft tissue structures made the inverse dynamic analysis more efficient and reliable. However, this made it less representative of real normal human anatomy [22].

## 5. Conclusion

Lower TFJ peak flexion and extension moments in ACLDM patients were found. During the middle and terminal stance phases, the ACLDM patients performed lower rectus femoris force than isolated ACL-deficient patients and the controls did, while no significant difference was found in hamstring force. Additionally, the gastrocnemius force in ACL-deficient patients both with and without a medial meniscus tear was lower than in the controls during the mid-stance phase. A concomitant medial meniscus tear could influence the muscular force patterns of ACLD patients. These results may help clinicians better understand the muscle function and gait pattern in ACL-deficient patients with a concomitant medial meniscus tear.

## Figures and Tables

**Table 1:** Subject characteristics in each group (mean ± SD).

	Control	ACLD	ACLDM	*P* value
Age (years)	29.3 ± 29.2	27.2 ± 3.8	27.2 ± 26.4	0.41
BMI (kg/m^2^)	25.2 ± 10.3	25.7 ± 12.2	24.6 ± 11.3	0.75
Pace (m/s)	1.25 ± 0.004	1.24 ± 0.007	1.24 ± 0.004	0.85
Time after injury (months)	n/a	10.2 ± 60.0	18.3 ± 46.8	0.27
Mechanism of injury	n/a	Sports injury (5 football, 6 basketball), 1 twisting injury while walking	Sports injury (2 football, 4 basketball), 2 twisting injury while walking, 2 injury while falling down the stairs	—
Anterior drawer test	n/a	All positive	All positive	—
Lachman test	n/a	All positive	All positive	—
Pivot shift test	n/a	8 positive, 4 negative	5 positive, 5 negative	0.43^∗^

BMI: body mass index; n/a: not applicable. ^∗^Chi-square test between ACLD and ACLDM groups.

**Table 2:** Maximal knee extension and flexion moment (Nm/kg) of knee flexors/extensors in ACLD and ACLDM groups.

	ACLD	ACLDM	*P* value
KT 2000 side-to-side difference (mm)	3.63 ± 1.85	4.32 ± 2.31	0.48
Flexor of unaffected side	0.91 ± 0.19	0.93 ± 0.28	0.77
Flexor of affected side	0.87 ± 0.23	0.67 ± 0.24	0.69
Extensor of unaffected side	1.65 ± 0.45	1.74 ± 0.72	0.77
Extensor of affected side	1.21 ± 0.44	1.05 ± 0.42	0.40

**Table 3:** Peak tibiofemoral moments and peak muscular forces of the lower limb.

	Control	ACLD	ACLDM
Peak flexion moment (nm/(kg^∗^m))	0.21 ± 0.23	0.16 ± 0.19	0.18 ± 0.10^∗^
Peak extension moment (nm/(kg^∗^m))	−0.23 ± 0.16	−0.14 ± 0.14	−0.11 ± 0.088^∗^
Peak GAS force (N/kg)	30.5 ± 14.1	29.2 ± 17.5	25.3 ± 9.8
Peak HAM force (N/kg)	15.6 ± 3.6	16.3 ± 3.5	15.5 ± 3.7
Peak RF force (N/kg)	4.4 ± 2.0	4.0 ± 1.5^△^	3.2 ± 0.74^∗^
Peak VAS force 1 (N/kg)	10.87 ± 6.81	9.39 ± 5.80	10.46 ± 4.18
Peak VAS force 2 (N/kg)	8.30 ± 7.84	9.88 ± 7.56	9.76 ± 4.32

PD: proximal-distal; AP: anterior-posterior; ML: medial-lateral; GAS: gastrocnemius; HAM: hamstring; RF: rectus femoris; VAS: vastus. Significant difference: ^∗^*P* < 0.05 between ACLDM and control groups. ^☆^*P* < 0.05 between ACLD and control groups. ^△^*P* < 0.05 between ACLD and ACLDM groups.

## Data Availability

The data used to support the findings of this study are available from the corresponding author upon request.

## References

[1] Majewski M., Susanne H., Klaus S. (2006). Epidemiology of athletic knee injuries: a 10-year study. *The Knee*.

[2] Rudolph K. S., Eastlack M. E., Axe M. J., Snyder-Mackler L. (1998). 1998 Basmajian Student Award Paper: movement patterns after anterior cruciate ligament injury: a comparison of patients who compensate well for the injury and those who require operative stabilization. *Journal of Electromyography and Kinesiology*.

[3] Roberts C. S., Rash G. S., Honaker J. T., Wachowiak M. P., Shaw J. C. (1999). A deficient anterior cruciate ligament does not lead to quadriceps avoidance gait. *Gait & Posture*.

[4] Papadonikolakis A., Cooper L., Stergiou N., Georgoulis A. D., Soucacos P. N. (2003). Compensatory mechanisms in anterior cruciate ligament deficiency. *Knee Surgery, Sports Traumatology, Arthroscopy*.

[5] Fuentes A., Hagemeister N., Ranger P., Heron T., de Guise J. A. (2011). Gait adaptation in chronic anterior cruciate ligament-deficient patients: pivot-shift avoidance gait. *Clinical Biomechanics*.

[6] Harato K., Niki Y., Kudo Y. (2015). Effect of unstable meniscal injury on three-dimensional knee kinematics during gait in anterior cruciate ligament-deficient patients. *The Knee*.

[7] Berchuck M., Andriacchi T. P., Bach B. R., Reider B. (1990). Gait adaptations by patients who have a deficient anterior cruciate ligament. *The Journal of Bone & Joint Surgery*.

[8] Shanbehzadeh S., Mohseni Bandpei M. A., Ehsani F. (2017). Knee muscle activity during gait in patients with anterior cruciate ligament injury: a systematic review of electromyographic studies. *Knee Surgery, Sports Traumatology, Arthroscopy*.

[9] Beard D. J., Soundarapandian R. R., Dodd C., O'Connor J. J. (1995). Gait and EMG analysis of anterior cruciate ligament deficient subjects. *Gait & Posture*.

[10] Messner K., Gao J. (1998). The menisci of the knee joint. Anatomical and functional characteristics, and a rationale for clinical treatment. *Journal of Anatomy*.

[11] Andernord D., Björnsson H., Petzold M. (2014). Surgical predictors of early revision surgery after anterior cruciate ligament reconstruction: results from the Swedish National Knee Ligament Register on 13,102 patients. *The American Journal of Sports Medicine*.

[12] Ahn J. H., Bae T. S., Kang K. S., Kang S. Y., Lee S. H. (2011). Longitudinal tear of the medial meniscus posterior horn in the anterior cruciate ligament-deficient knee significantly influences anterior stability. *The American Journal of Sports Medicine*.

[13] Hosseini A., Li J.-S., Gill T. J., Li G. (2014). Meniscus injuries alter the kinematics of knees with anterior cruciate ligament deficiency. *Orthopaedic Journal of Sports Medicine*.

[14] Zhang Y., Huang W., Ma L., Lin Z., Huang H., Xia H. (2015). Kinematic characteristics of anterior cruciate ligament deficient knees with concomitant meniscus deficiency during ascending stairs. *Journal of Sports Sciences*.

[15] Hall M., Bryant A. L., Wrigley T. V. (2016). Does meniscal pathology alter gait knee biomechanics and strength post-ACL reconstruction?. *Knee Surgery, Sports Traumatology, Arthroscopy*.

[16] Xu Y., Ao Y. (2002). Clinical study on the meniscal injury following the rupture of anterior cruciate ligament of the knee. *Chinese Journal of Orthopaedics*.

[17] Outerbridge R. E., Outerbridge H. (2001). The etiology of chondromalacia patellae. *Clinical Orthopaedics and Related Research*.

[18] Kadaba M. P., Ramakrishnan H. K., Wootten M. E. (1990). Measurement of lower extremity kinematics during level walking. *Journal of Orthopaedic Research*.

[19] Damsgaard M., Rasmussen J., Christensen S. T., Surma E., de Zee M. (2006). Analysis of musculoskeletal systems in the AnyBody Modeling System. *Simulation Modelling Practice and Theory*.

[20] Horsman M. D. K., Koopman H. F. J. M., van der Helm F. C. T., Prosé L. P., Veeger H. E. J. (2007). Morphological muscle and joint parameters for musculoskeletal modelling of the lower extremity. *Clinical Biomechanics*.

[21] Rasmussen J., Damsgaard M., Voigt M. (2001). Muscle recruitment by the min/max criterion - a comparative numerical study. *Journal of Biomechanics*.

[22] Andersen M. S., Benoit D. L., Damsgaard M., Ramsey D. K., Rasmussen J. (2010). Do kinematic models reduce the effects of soft tissue artefacts in skin marker-based motion analysis? An in vivo study of knee kinematics. *Journal of Biomechanics*.

[23] CL Vaughan B. D., O'Connor J. C. (1992). *Dynamics of human gait*.

[24] Wellsandt E., Gardinier E. S., Manal K., Axe M. J., Buchanan T. S., Snyder-Mackler L. (2016). Decreased knee joint loading associated with early knee osteoarthritis after anterior cruciate ligament injury. *The American Journal of Sports Medicine*.

[25] Andriacchi T. P., Koo S., Scanlan S. F. (2009). Gait mechanics influence healthy cartilage morphology and osteoarthritis of the knee. *The Journal of Bone & Joint Surgery*.

[26] Zabala M. E., Favre J., Scanlan S. F., Donahue J., Andriacchi T. P. (2013). Three-dimensional knee moments of ACL reconstructed and control subjects during gait, stair ascent, and stair descent. *Journal of Biomechanics*.

[27] Noyes F. R., Schipplein O. D., Andriacchi T. P., Saddemi S. R., Weise M. (1992). The anterior cruciate ligament-deficient knee with varus alignment: an analysis of gait adaptations and dynamic joint loadings. *The American Journal of Sports Medicine*.

[28] Ferber R., Osternig L. R., Woollacott M. H., Wasielewski N. J., Lee J.-H. (2002). Gait mechanics in chronic ACL deficiency and subsequent repair. *Clinical biomechanics*.

[29] Knoll Z., Kocsis L., Kiss R. M. (2004). Gait patterns before and after anterior cruciate ligament reconstruction. *Knee Surgery, Sports Traumatology, Arthroscopy*.

[30] Torry M. R., Decker M. J., Ellis H. B., Shelburne K. B., Sterett W. I., Steadman J. R. (2004). Mechanisms of compensating for anterior cruciate ligament deficiency during gait. *Medicine & Science in Sports & Exercise*.

[31] Frank R. M., Lundberg H., Wimmer M. A. (2016). Hamstring activity in the anterior cruciate ligament injured patient: injury implications and comparison with quadriceps activity. *Arthroscopy*.

[32] Fleming B. C., Renstrom P. A., Ohlen G. (2001). The gastrocnemius muscle is an antagonist of the anterior cruciate ligament. *Journal of Orthopaedic Research*.

[33] Adouni M., Shirazi-Adl A., Marouane H. (2016). Role of gastrocnemius activation in knee joint biomechanics: gastrocnemius acts as an ACL antagonist. *Computer Methods in Biomechanics and Biomedical Engineering*.

